# Editorial Note: Towards secure and efficient integration of blockchain and 6G networks

**DOI:** 10.1371/journal.pone.0350253

**Published:** 2026-05-28

**Authors:** The PLOS One Editors

The *PLOS One* Editors issue this Editorial Note because this article [1] was identified as one of a series of submissions for which we have concerns about aspects of the peer review process. Readers are advised to interpret the article [1] with caution.

The Editors have no evidence of any author involvement in the peer review concerns.

## References

[1] Liu Y, Peng S, Zhang M, Shi S, Fu J. Towards secure and efficient integration of blockchain and 6G networks. PLoS One. 2024;19(4):e0302052. doi: 10.1371/journal.pone.0302052 38603725 PMC11008884

